# For money or service? a cross-sectional survey of preference for financial versus non-financial rural practice characteristics among ghanaian medical students

**DOI:** 10.1186/1472-6963-11-300

**Published:** 2011-11-03

**Authors:** Jennifer C Johnson, Emmanuel Nakua, Mawuli Dzodzomenyo, Peter Agyei-Baffour, Mawuli Gyakobo, Kwesi Asabir, Janet Kwansah, S Rani Kotha, Rachel C Snow, Margaret E Kruk

**Affiliations:** 1 University of Michigan Center for Global Health, Galleria Building, 1214 S. University Ave, 2nd Floor Suite C, Ann Arbor, Michigan 48104 USA; 2 Kwame Nkrumah University of Science and Technology, Department of Community Health, PMB, Kumasi, Ghana, West Africa; 3 University of Ghana School of Public Health, Department of Biological, Environmental and Occupational Health Sciences, PO Box LG 13, University of Ghana, Legon, Ghana; 4 Kwame Nkrumah University of Science and Technology, Department of Community Health, Kumasi, Ghana; 5 University of Ghana, c/o Office of the Provost, College of Health Sciences, University of Ghana, PO Box KB 52, Korle Bu, Ghana; 6 Ministry of Health, Human Resource for Health Directorate, P.O.Box M44, Accra, Ghana; 7 Ministry of Health, Policy, Planning, Monitoring, and Evaluation Directorate, PO Box M44, Accra, Ghana; 8 University of Michigan Center for Global Health, Galleria Building, 1214 S. University Ave, 2nd Floor Suite C, Ann Arbor, Michigan 48104 USA; 9 University of Michigan School of Public Health, Department of Health Behavior and Health Education, 1415 Washington Heights 3814 SPH I, Ann Arbor, Michigan 48109 USA; 10 Columbia University Mailman School of Public Health, 600 W. 168th Street, Room 606, New York, NY 10032

**Keywords:** human resources for health, Ghana, rural practice, emigration, rural incentives, medical doctors

## Abstract

**Background:**

Health worker shortage and maldistribution are among the biggest threats to health systems in Africa. New medical graduates are prime targets for recruitment to deprived rural areas. However, little research has been done to determine the influence of workers' background and future plans on their preference for rural practice incentives and characteristics. The purpose of this study was to identify determinants of preference for rural job characteristics among fourth year medical students in Ghana.

**Methods:**

We asked fourth-year Ghanaian medical students to rank the importance of rural practice attributes including salary, infrastructure, management style, and contract length in considering future jobs. We used bivariate and multivariate ordinal logistic regression to estimate the association between attribute valuation and students' socio-demographic background, educational experience, and future career plans.

**Results:**

Of 310 eligible fourth year medical students, complete data was available for 302 students (97%). Students considering emigration ranked salary as more important than students not considering emigration, while students with rural living experience ranked salary as less important than those with no rural experience. Students willing to work in a rural area ranked infrastructure as more important than students who were unwilling, while female students ranked infrastructure as less important than male students. Students who were willing to work in a rural area ranked management style as a more important rural practice attribute than those who were unwilling to work in a rural area. Students studying in Kumasi ranked contract length as more important than those in Accra, while international students ranked contract length as less important than Ghanaian students.

**Conclusions:**

Interventions to improve rural practice conditions are likely to be more persuasive than salary incentives to Ghanaian medical students who are willing to work in rural environments a priori. Policy experiments should test the impact of these interventions on actual uptake by students upon graduation.

## Background

Health workers are a critical element to improving global health [1]. One of the biggest challenges to health systems is to, "get the right workers with the right skills in the right place doing the right things, " [2]. Yet the availability of medical services continues to vary inversely with need, according to the Inverse Care Law [3]. Today's 60 million health workers are maldistributed, with a strong bias favoring wealthy countries and urban areas.

A number of factors pull health workers to urban areas in low- and middle- income countries (LAMICs). Urban areas offer more career and educational opportunities; better working conditions, equipment, and infrastructure; easier access to private practice; concentrated services and amenities; more diverse leisure activities; and better employment prospects for spouses and schools for children [4-6]. Rural populations often suffer from higher poverty and lack of safe drinking water and sanitation, contributing to a poorer general health status and greater need [7].

Strategies to recruit and retain rural health workers vary widely, including education reforms (e.g. recruitment of rural students, conducting training programs in rural areas), professional and personal support (e.g. provisions for housing, training opportunities), financial incentives, and compulsory service [4,6-11]. Evidence of the impact of these strategies on health worker distribution in LAMICs is poor, due largely to difficulty in collecting data and designing rigorous evaluation [7,11,12]. Health sector reforms are hindered by limited institutional capacity of governments, structural obstacles within the health system, lack of legislation to back up reform policies, ineffective inter-sectoral collaboration, and resistance to change, which challenge the systematic implementation of policy change and evaluation of impact [13,14]. Nonetheless, research on financial and non-financial incentives for deprived-area practice have been identified by policy-makers as having a high potential for health impact [15].

There are several methods for studying the importance of incentives to health care workers. In settings where rigorous evaluation of human resource distribution is not possible, stated preference studies or discrete choice experiments (DCEs) provide insight into health worker motivations to inform policy [16]. The discrete choice method assumes that job postings can be described by their attributes and that the decisions of individuals are a function of the total utility gained by these attributes.

Previous stated preference studies in low-income countries have examined the impact of incentives related to: deprived area allowance or salary top up; workload; availability of equipment and drugs; facility infrastructure; contract length; housing; transportation; educational opportunities and training; promotion; and management and supervision [5,17-22]. Most studies have shown considerable heterogeneity in incentive preference. However, attempts to explain preference heterogeneity have generally focused on demographic characteristics (e.g. sex, marital status, parenthood) [20-22]. We have found no studies examining the association between medical students' future career plans and their valuation of different job characteristics.

In addition to the job attributes, background factors have an impact on individual decision-making. Health workers and students of a rural origin, "underserved" ethnic backgrounds, low socioeconomic background, male sex, young age, single marital status, and those with an interest in rural practice at study entry are more likely to practice in a rural area after graduation, although these relationships vary across settings [6,7,10,23].

Ghana is a low-income country in West Africa with a critical shortage of health care providers [2]. Between 1985 and 1994, 61% of medical school graduates emigrated, primarily to the UK and the USA [23]. Although the health sector currently lacks a reliable information management system, a recent Ministry of Health report indicates a possible decline in emigration since 2006 [24]. Of the nearly 2500 physicians practicing in Ghana [25], over 70% are located in urban areas [26], despite the fact that two-thirds of Ghanaians live in rural areas [27]. Recruitment of doctors to rural postings is a serious challenge; for example, of the 43 doctors posted to the rural Upper East Region from 2001-2009, only 4 assumed their posts [28]. Inequitable distribution of health workers is noted as a key challenge in the Ministry of Health's (MoH) 2009 Programme of Work [28].

The Ghana MoH has implemented a number of incentives aimed at recruiting and retaining health staff in the country and deprived areas. These include a 20-30% salary top up for health staff in deprived areas implemented from 2004 and a staff vehicle purchase scheme since 1997. A total of about 2900 health staff have benefited from this scheme, including over 1000 medical officers. In 2005, the Ghana College of Physicians and Surgeons was established and anecdotal evidence suggests a marked reduction in emigration. The selection of residents into the college is also currently being revised with a deprived area focus. For example, scholarship packages may soon be offered to medical officers who are working in rural areas.

Financial and non-financial incentives have different costs and regulatory implications for governments - the main payers for health services in sub-Saharan Africa. Understanding the effect of prior interest (or lack thereof) in rural practice on students' valuation of different job characteristics would enhance efficiency of funds spent on incentive packages. This paper aims to identify individual factors that are associated with preference for salary versus non-salary job characteristics for rural positions among fourth year medical students in Ghana.

## Methods

### Study Population

We surveyed Ghanaian medical students in their fourth year. These students had experienced the clinical environment, but not yet made decisions about job placements. At the time of the study, three Ghanaian medical schools enrolled fourth year students: the University of Ghana (UG), Kwame Nkrumah University of Science and Technology (KNUST), and University for Development Studies (UDS). We invited all fourth-year medical students at these schools in May 2009 to participate; no sampling was conducted.

We obtained ethics approval from the Ghana Health Service Ethical Review Committee; the UG Medical School; the KNUST Committee on Human Research, Publications, and Ethics; and the University of Michigan Institutional Review Board. We obtained informed consent from all respondents prior to their participation. Other manuscripts have previously reported on the methods of this study [21].

### Variables and questionnaire administration

The survey collected information about respondents' demographic background, rural and international living experience, and future career plans. We based demographic questions on the latest Ghana Demographic and Health Survey. The survey was administered in on-campus computer laboratories using Sawtooth Software CAPI software, with trained facilitators available to answer questions at all times. Facilitated focus group discussions with third- and fifth-year medical students at UG and KNUST informed the design of the preference module of the survey, which also included a discrete choice experiment (DCE, see Kruk et al for more details [21]).

To obtain the dependent variable, we asked students to directly rank the importance of rural job attributes on a scale from 1-7, with one being the most important. The attributes were Salary (base salary to 2× base salary); Allowance for Children's Education; Infrastructure Equipment, and Supplies (basic versus advanced); Management Style (supportive versus unsupportive); Contract Length (2 versus 5 years); Housing (basic versus superior housing); and Transportation (utility car). In this exercise, each numeric rank could only be assigned to one attribute. We restricted this analysis to four attributes identified as the most important during the DCE: Salary; Infrastructure, Equipment, and Supplies; Management Style; and Contract Length [21]. The importance rank (1-7) given to each rural attribute was the dependent variable in our analysis.

For the independent variables, in addition to students' demographic and socioeconomic characteristics, we collected information about students' preferences for rural practice, career aspirations, and preferred rural incentives and conditions. The key independent variables of interest were: school, rural vs urban origin, and willingness to undertake rural work after graduation.

### Statistical analysis

We used ordinal logistic regression (OLR), a statistical technique designed for data with an ordered dependent variable. OLR models the cumulative probabilities of being in higher ordered categories, compared with being in all lower (pooled) categories, thus simultaneously estimating n-1 equations for an outcome with a total of n levels. This allows us to present the odds of ranking an attribute at a certain importance level, compared to lower levels in a single odds ratio (OR).

Initial analysis with attributes ranked from 1-7 suggested that this ranking violated the proportional odds assumption that the relationship between each pair of outcome groups is the same. Thus, we collapsed numeric rankings into approximate tertiles representing high, medium, and low importance; none of these analyses violated the proportional odds assumption.

Predictors of interest included: university attended, sex, age group, ethnicity, rural living experience, family socioeconomic background (SES), secondary school, student enrollment type, willingness to work in a rural area, and consideration of emigration.

Although UDS students represented a small proportion of this population, we included them in this analysis because UDS is located in a more rural area in northern Ghana. University of Ghana and KNUST are in the larger cities of Accra and Kumasi, respectively. We wanted to know if these students' preferences would differ in a systematic way. We examined the impact of Ga/Dangme, Ewe, and minority ethnic backgrounds, with Akan being the referent. We determined family SES by combining maternal and paternal education level and profession. If one or more parent had attended University and was employed in "Professional or managerial work (e.g. doctor, lawyer, engineer, accountant), " then the family was defined as high-SES. Student enrollment type included those who were sponsored by the government, those paying fees, and international students. For the regression analyses, we transformed willingness to work in a rural area into a dichotomous variable, comparing those who responded "I will definitely work in a deprived area"/"I am likely to work in a deprived area" to those who responded "I will definitely not work in a deprived area"/"I am unlikely to work in a deprived area."

We used bivariate and multivariate ordinal logistic regression using sociodemographic characteristics and career plans to predict self-ranked attribute importance, separately for each of the four most important attributes. We used Stata 10.1 for all statistical analyses, including the user-written command omodel [29].

## Results

Out of 310 fourth-year medical students enrolled in Ghana's medical schools, 307 participated in the survey (99%). Five surveys were corrupted by viruses or lost due to technological malfunction, making the final analysis dataset 302 individuals. The survey took a mean of 31.6 minutes (SD 12.45).

Table 1 shows the students' sociodemographic characteristics and career plans. There were more male students than female (183 and 118, respectively). Over three-quarters of students lacked rural living experience (76.1%) and over half of the students came from a high SES family (58.8%). Over half of students stated they were *likely to *OR *definitely will *work in a rural area (49% and 6.6%, respectively), while over two-thirds had contemplated emigration after graduating (68.1%). The proportions of students in each attribute importance category (high, medium, low) were set to most closely approximate tertiles.

**Table 1:** Background characteristics of respondents (N = 302)

	N (%)^1^
University	
UG	179 (59.3)
UST	111 (36.8)
UDS	12 (4.0)

Sex	
Female	118 (39.2)
Male	183 (60.8)

Age group	
22 and down	120 (40.4)
23-24	150 (50.5)
25 and up	27 (9.1)

Ethnicity	
Akan	137 (46.0)
Ga/Dangme	40 (13.4)
Ewe	39 (13.1)
Other	82 (27.5)

Rural living experience	
Yes	72 (23.9)
No	229 (76.1)

Family SES^2^	
High	173 (58.8)
Low	121 (41.2)

Secondary School (SS)	
Top-tier^3^	82 (27.5)
Other	216 (72.5)

Student type	
Sponsored	214 (72.3)
Fee-Paying	42 (14.2)
International	40 (13.5)

Willingness to work in a rural area	
Definitely will not	19 (6.6)
Unlikely	108 (37.8)
Likely	140 (49.0)
Definitely will	19 (6.6)

Considered emigration	
Yes	186 (68.1)
No	87 (31.9)

Importance of Salary^4^	
High	131 (43.5)
Medium	112 (37.2)
Low	58 (19.3)

Importance of Infrastructure^4^	
High	102 (33.9)
Medium	113 (37.5)
Low	86 (28.6)

Importance of Management Style^4^	
High	61 (20.3)
Medium	108 (36.0)
Low	131 (43.7)

Importance of Contract Length^4^	
High	87 (28.9)
Medium	77 (25.6)
Low	137 (45.5)

1 N may not add to 302 due to missing responses; percentages have been adjusted

2 High family SES includes students where the mother and/or father is a University-trained professional

3 Top-tier secondary schools include those in Ghana Education Service Category A (ranked based on facilities)

4 Based on a 7-unit scale; categorization of attribute importance most closely approximates tertiles

Table 2 shows the results of the bivariate analysis. The results are discussed below by attribute.

**Table 2:** Bivariate ordinal regression of self-ranked attribute importance and individual characteristics.

	Salary OR (95% CL)			Infrastructure OR (95% CL)			Management OR (95% CL)			Contract Length OR (95% CL)		
Female	0.75	(0.49,	1.16)	0.69	(0.45,	1.06)	0.98	(0.63,	1.52)	0.75	(0.49,	1.16)

University												
UG	Ref			Ref			Ref			Ref		
UST	1.44	(0.92,	2.25)	1.02	(0.66,	1.57)	0.73	(0.47,	1.14)	1.46	(0.94,	2.27)
UDS	3.51	(1.06,	11.65)	1.28	(0.46,	3.62)	0.75	(0.26,	2.13)	3.47	(1.18,	10.24)

Age group												
22 and younger	0.68	(0.31,	1.48)	1.64	(0.76,	3.55)	1.69	(0.79,	3.64)	0.44	(0.20,	0.99)
23-24	0.55	(0.25,	1.20)	2.02	(0.94,	4.32)	1.48	(0.70,	3.14)	0.47	(0.22,	1.04)
25 and older	Ref			Ref			Ref			Ref		

Ethnicity												
Akan	Ref			Ref			Ref			Ref		
Ga/Dangme	0.90	(0.46,	1.75)	1.21	(0.63,	2.33)	1.14	(0.58,	2.24)	0.81	(0.41,	1.60)
Ewe	0.39	(0.20,	0.75)	0.68	(0.34,	1.35)	1.57	(0.79,	3.09)	1.27	(0.64,	2.51)
Other	0.89	(0.53,	1.49)	1.09	(0.66,	1.80)	0.89	(0.54,	1.48)	1.21	(0.73,	2.00)

Lived in a rural area	0.58	(0.35,	0.97)	1.24	(0.76,	2.03)	0.86	(0.53,	1.40)	1.65	(1.00,	2.73)

High family SES^1^	1.39	(0.90,	2.15)	1.16	(0.75,	1.77)	0.83	(0.53,	1.28)	0.86	(0.56,	1.33)

Attended a top SS	1.35	(0.84,	2.17)	0.75	(0.46,	1.21)	0.84	(0.52,	1.35)	1.04	(0.64	1.68)

Student type												
Sponsored	Ref			Ref			Ref			Ref		
Fee-paying	0.53	(0.28,	1.00)	1.09	(0.58,	2.04)	1.93	(1.01,	3.66)	0.73	(0.40,	1.35)
International	0.93	(0.50,	1.72)	1.98	(1.05,	3.73)	1.11	(0.60,	2.04)	0.48	(0.25,	0.92)

Willing to work rural^2^	0.80	(0.52,	1.25)	1.59	(1.03,	2.46)	1.59	(1.02,	2.47)	1.00	(0.65,	1.54)

Considered Emigration^3^	1.76	(1.09,	2.84)	1.06	(0.66,	1.69)	0.69	(0.43,	1.11)	0.74	(0.46,	1.21)

1 High SES defined as mother or father is a University-trained professional

2 "Likely to" or "definitely will" work in a rural area after graduation, compared to "definitely will not" or "unlikely to" work in a deprived area after graduation.

3 Has considered emigrating to another country after graduation

***• Salary: ***UDS students and those considering emigration ranked salary as more important than UG students and those not considering emigration [OR 3.51 (1.06-11.65); OR 1.76 (1.09, 2.84)]. Ewe students and those with rural living experience students ranked salary as less important than Akan students and those without rural experience [OR 0.39 (0.20-0.75); OR 0.58 (0.35, 0.97)]. Fee-paying students ranked salary as less important than sponsored students [OR 0.53 (0.28-1.00)].

***• Infrastructure:***International students ranked infrastructure as more important than Ghanaian students [1.98 (1.05-3.73)]. Those willing to work in a rural area ranked infrastructure as more important than those unwilling to work in a rural area [OR 1.59 (1.03-2.46)].

***• Management style: ***Fee-paying students ranked management style as more important than sponsored students [OR 1.93 (1.01-3.66)]. Those willing to work in a rural area ranked management style as more important than those unwilling to work in a rural area [OR 1.59 (1.02-2.47)].

***• Contract Length: ***UDS students and those with rural experience ranked reduced contract length as a more important rural practice attribute than UG students and those without rural experience [OR 3.47 (1.18-10.24); OR 1.65 (1.00-2.73)]. Younger students and international students ranked reduced contract length as less important than older students and Ghanaian students [OR 0.44 (0.20-0.99); OR 0.48 (1.00-2.73)].

Table 3 shows the results of the multivariate analysis with all sociodemographic factors and career plans. The results are discussed below by attribute.

**Table 3:** Multivariate ordinal regression of self-ranked attribute importance and individual characteristics.

	Salary OR (95% CL)			Infrastructure OR (95% CL)			Management Style OR (95% CL)			Contract Length OR (95% CL)		
Female	0.68	(0.39,	1.20)	0.54	(0.31,	0.93)	0.82	(0.47,	1.43)	0.79	(0.45,	1.37)

University												
UG	Ref			Ref			Ref			Ref		
UST	1.22	(0.68,	2.18)	1.07	(0.61,	1.89)	0.80	(0.45,	1.42)	1.96	(1.10,	3.50)
UDS	3.68	(0.71,	18.93)	4.91	(0.99,	24.37)	3.60	(0.74,	17.61)	1.24	(0.26,	5.91)

Age group												
22 and younger	0.51	(0.17,	1.58)	3.38	(1.07,	10.68)	3.38	(1.05,	10.91)	0.54	(0.18,	1.66)
23-24	0.57	(0.19,	1.72)	4.10	(1.33,	12.69)	2.35	(0.75,	7.34)	0.49	(0.16,	1.44)
25 and older	Ref			Ref			Ref			Ref		

Ethnicity												
Akan	Ref			Ref			Ref			Ref		
Ga/Dangme	0.74	(0.35,	1.56)	1.16	(0.56,	2.40)	1.03	(0.48,	2.19)	0.99	(0.47,	2.10)
Ewe	0.39	(0.18,	0.87)	0.56	(0.25,	1.26)	1.44	(0.65,	3.17)	1.64	(0.74,	3.64)
Other	0.68	(0.28,	1.64)	0.66	(0.28,	1.59)	0.62	(0.25,	1.50)	1.94	(0.83,	4.52)

Lived in a rural area	0.45	(0.24,	0.84)	1.33	(0.72,	2.46)	0.70	(0.38,	1.28)	1.34	(0.74,	2.42)

High family SES^1^	1.15	(0.66,	2.01)	0.94	(0.55,	1.59)	0.89	(0.51,	1.55)	1.14	(0.67,	1.97)

Attended a top SS	1.14	(0.56,	2.32)	1.03	(0.52,	2.07)	0.77	(0.38,	1.56)	0.68	(0.34,	1.39)

Student Type												
Sponsored	Ref			Ref			Ref			Ref		
Fee-paying	0.62	(0.28,	1.38)	1.35	(0.61,	3.00)	2.32	(1.04,	5.19)	0.84	(0.39,	1.84)
International	1.08	(0.33,	3.52)	4.45	(1.32,	15.00)	1.94	(0.58,	6.56)	0.27	(0.08,	0.90)

Willing to work rural^2^	0.89	(0.53,	1.52)	1.77	(1.06,	2.97)	1.86	(1.09,	3.17)	0.70	(0.41,	1.19)

Considered emigration^3^	1.93	(1.11,	3.36)	0.94	(0.55,	1.61)	0.65	(0.37,	1.13)	0.90	(0.52,	1.56)

1 High SES defined as mother or father is a University-trained professional

2 "Likely to" or "definitely will" work in a rural area after graduation, compared to "definitely will not" or "unlikely to" work in a deprived area after graduation.

3 Has considered emigrating to another country after graduation

***• Salary: ***Students who have considered emigration ranked salary as more important than those not considering emigration [OR 1.93 (1.11-3.36)]. Ewe students and those with rural living experience ranked salary as a less important rural practice attribute than Akan students and those without rural living experience [OR 0.39 (0.18-0.87); OR 0.45 (0.24-0.84)].

***• Infrastructure: ***Younger students and international students ranked infrastructure as more important than older students and Ghanaian students [under 23 years old: OR 3.38 (10.7-10.68); 23-24 years old OR: 4.10 (1.33-12.69); international OR 4.45(1.32-15.00)]. Those willing to work in a rural area ranked infrastructure as more important than those unwilling to work in a rural area [OR 1.77 (1.06-2.97), ]. Female students ranked infrastructure as a less important rural practice attribute than male students [OR 0.54 (0.31-0.93)].

***• Management Style: ***Younger students and those paying school fees ranked management style as a more important rural practice attribute than older students and sponsored students [OR 3.38 (1.05-10.91); OR 2.32 (10.4-5.19)]. Those willing to work in a rural area ranked management style as a more important rural practice attribute than those unwilling to work in a rural area [OR 1.86 (1.09-3.17)]

***• Contract length: ***KNUST students ranked reduced contract length as a more important attribute to rural practice than UG students [OR 1.96 (1.10-3.50)]. International students ranked contract length as less important than Ghanaian students [OR 0.27 (0.08-0.90)].

## Discussion

In a study of 302 fourth-year medical students in Ghana, we found that students' demographic characteristics and career plans were associated with preference for rural practice incentives and conditions. In particular, students who stated a willingness to work in a rural area after graduation were more likely to rank non-financial conditions (infrastructure and management style) as more important than those who were unwilling to work in a rural area, while students who considered emigration had a greater importance for salary than students not considering emigration. Compared with higher-income countries, physician salaries in Ghana are very low - equivalent to approximately 1, 100 USD per month in 2008 [30]. While rural and urban physicians receive the same base salary, the Ghana MoH provides rural officers an additional deprived area incentive. The MoH does this in an attempt to compensate for lost locum opportunities, which are almost exclusively in urban areas and represent a substantial income supplement.

We also established that men, younger students, and international students placed a higher value on hospital infrastructure (equipment, supplies, medicines) than women, older students, and Ghanaian-born students. A recent study of practicing doctors in Ethiopia also found an association between salary preference and sex and age [20]. However, this study examined incentives for both urban and rural postings, while the current study was specifically targeted to rural practice, which could help explain the differences in findings regarding salary preference. These two studies together provide evidence of an impact of individual sociodemographic background on preferred rural practice incentives and conditions. Qualitative research should be undertaken to determine the reasons why sociodemographic background should impact preference for rural incentives.

Reduced contract length before study leave is a particularly relevant incentive being discussed by policy makers in Ghana and other low-income countries. Interestingly, in the multivariate model KNUST students studying in Kumasi found the reduced contract length more persuasive than UG students studying in the larger city of Accra. This could be related to a greater lifetime exposure to rural living for UG students, compared to KNUST students (23.5% vs. 19.8%, χ^2 ^= 13.82, p = 0.008). Students without previous experience in a rural area may be more concerned about a speedy and reliable exit than those who are more comfortable in a rural setting. In addition, international students ranked reduced contract length as less important than Ghanaian students. This is not surprising given international students are more likely to have contemplated emigration (94.6% vs. 63.4%, χ^2 ^= 14.22, p < 0.001) and less willing to work in a rural area (34.2% vs. 59.3%, χ^2 ^= 8.36, p = 0.004), in general.

The association between future career plans and attribute importance is novel. It is possible that those who stated willingness for rural practice ranked attributes according to their anticipated needs (i.e. they were placing themselves in the shoes of a rural doctor), while those who were unwilling ranked attributes in an effort to explain their lack of willingness for rural practice (i.e. they were never seriously considering the posts). This study points to two groups of students with different likelihood of accepting a rural posting and associated attribute preferences.

These results have implications for policies designed to increase rural recruitment of new graduates. Many of the fourth-year students, two years away from graduation, are already opposed to working in a rural environment. In addition, the majority of students are contemplating emigration. Students who intend to emigrate and those who are opposed to rural work are unlikely to be susceptible to policies aimed at incentivizing rural practice. Rather than widely targeting the preferences of all graduating students, recruitment policies could specifically target those students who are willing to work in a rural area a priori. This rationale suggests a greater emphasis on non-financial conditions that enable physicians to perform well clinically and enable their professional growth, as opposed to salary top-ups. The results reinforce the finding that non-financial incentives were highly valued by medical students in Ghana [21].

This study has several limitations. First, the study population was young and fairly naïve to the conditions to rural practice. Less than a quarter of students surveyed had any rural living experience and only 60% of students had participated in rural outreach or service in a deprived area during medical school. After gaining clinical experience in a rural area, health workers preferences for rural conditions and incentives are likely to change. In addition, less than one percent of students surveyed were married. As health workers age, their preferences may shift as they start families and look for more permanent living situations.

We selected a student population for this study, instead of practicing physicians, because recent medical school graduates are the main targets of rural physician recruitment by the Ghana MoH. Moreover, surveying students allowed us to capture both those who will stay and work in Ghana as well as those who will emigrate shortly after graduation - the latter would be lost in a survey of practicing physicians in Ghana. Understanding these students' motivations and perspectives is key to solving the HRH crisis in Ghana. Nonetheless, this study population choice limits the generalizability of these results to young students and recent graduates from medical school. Future studies should quantify the preferences of practicing physicians to inform policies aimed at retention and/or recruitment of senior physicians to rural areas.

In addition, social desirability bias may have influenced students' responses to the survey questions. Many focus group participants expressed feeling a high social pressure to work in a deprived area. In addition, students may have felt pressure to de-emphasize the importance of financial incentives. To attempt to correct for social desirability, students were informed that they could skip any question they did not want to answer and were assured of the confidentiality of their responses. Lastly, this study investigated stated preferences and did not follow participants over time to validate results against actual decision-making. Ideally, policy experiments informed by research would test the impact of interventions to promote uptake of rural posts.

In a qualitative study conducted with Ghanaian doctors and medical leaders from both urban and rural environments, Snow et al found that rural physicians were mostly self-described adventurers, locals who had returned home to work, or idealists motivated by mission or ideology [12]. These studies combined suggest that those physicians who end up practicing in rural areas are a specific subset of all practicing workers in Ghana, with distinct preferences and needs.

## Conclusions

Ghanaian medical students who have considered emigration value a rural practice salary incentive more than students who have not considered emigration. Students who are willing to practice in a rural area value infrastructure and management style more than students who are unwilling to practice in a rural area. Improvements in rural practice conditions are likely to be more persuasive to those willing to work in rural environments a priori. This approach may be more cost-effective for the Ghanaian government if the incentive structure is better matched to students who are more likely to take up rural opportunities. Policy experiments should test the impact of these interventions on actual uptake by Ghanaian medical students upon graduation.

## List of Abbreviations

DCE: Discrete Choice Experiment; MoH: Ghana Ministry of Health; KNUST: Kwame Nkrumah University of Science and Technology; OR: Odds ratio; OLR: Ordinal logistic regression; SES: Socioeconomic Status/Background); UDS: University for Development Studies; UG: University of Ghana.

## Competing interests

The authors declare that they have no competing interests.

## Authors' contributions

JCJ, EN, MD, PAB, MG, KA, JK, SRK, RCS, and MEK jointly conceived the study and designed the survey. JCJ, MG, PAB, EN, and KA carried out data collection, under supervision by MEK. All authors were involved in the interpretation of study findings. JCJ wrote the first draft of the manuscript. All authors reviewed and critically revised the manuscript for important intellectual content and agreed to submit the manuscript for publication.

## Pre-publication history

The pre-publication history for this paper can be accessed here:

http://www.biomedcentral.com/1472-6963/11/300/prepub
